# Supplementary material for: Cost profiles of cancer patients at the end of life: Estimates from the EPICOST-study
Source: PLoS One. 2025 Feb 14;20(2):e0318849. doi: 10.1371/journal.pone.0318849 (PMC11828425; doi:10.1371/journal.pone.0318849)
Supplement: S1 Table — (DOCX) [file pone.0318849.s001.docx]

**S1 Table. Annual average costs, complete prevalent cases and total costs in the final phase by cancer type and health care services database.**

| **Cancer type** | **Health care service database** | **Average cost (€)** | **Prevalent cases** | **Total cost (€)** |
| --- | --- | --- | --- | --- |
| Colon | md | 215.87 | 1,001 | 216,086.07 |
|  | lrc | 628.33 | 1,001 | 628,962.32 |
|  | hd | 2,877.20 | 1,001 | 2,880,075.82 |
|  | dp | 326.31 | 1,001 | 326,640.79 |
|  | hsp | 1,114.35 | 1,001 | 1,115,460.91 |
|  | er | 787.77 | 1,001 | 788,553.75 |
|  | ha | 9,259.44 | 1,001 | 9,268,700.91 |
|  | ihc | 712.55 | 1,001 | 713,260.57 |
|  | ops | 2,687.96 | 1,001 | 2,690,645.02 |
| Breast | md | 92.44 | 1,356 | 125,351.56 |
|  | lrc | 607.18 | 1,356 | 823,336.70 |
|  | hd | 2,423.89 | 1,356 | 3,286,789.99 |
|  | dp | 467.86 | 1,356 | 634,422.97 |
|  | hsp | 1,116.62 | 1,356 | 1,514,129,97 |
|  | er | 650.87 | 1,356 | 882,580.45 |
|  | ha | 5,277.33 | 1,356 | 7,156,057.22 |
|  | ihc | 638.66 | 1,356 | 866,018.35 |
|  | ops | 1,631.34 | 1,356 | 2,212,093.30 |
| Melanoma | md | 87.96 | 275 | 24,188.01 |
|  | lrc | 229.16 | 275 | 63,018.42 |
|  | hd | 14,992.48 | 275 | 4,122,932.95 |
|  | dp | 264.90 | 275 | 72,848.82 |
|  | hsp | 431.23 | 275 | 118,588.27 |
|  | er | 1,014.57 | 275 | 279,006.11 |
|  | ha | 8,859.28 | 275 | 2,436,302.21 |
|  | ihc | 714.32 | 275 | 196,436.84 |
|  | ops | 707.98 | 275 | 194,694.38 |
| Rectum | md | 761.13 | 446 | 339,464.34 |
|  | lrc | 465.16 | 446 | 207,460.13 |
|  | hd | 3,407.06 | 446 | 1,519,549.70 |
|  | dp | 499.29 | 446 | 222,685.50 |
|  | hsp | 956.81 | 446 | 426,736.79 |
|  | er | 788.88 | 446 | 351,842.25 |
|  | ha | 8,918.65 | 446 | 3,977,718.30 |
|  | ihc | 1,034.10 | 446 | 461,209.16 |
|  | ops | 2,890.76 | 446 | 1,289,278.92 |
| Thyroid | md | 33.34 | 79 | 2,633.56 |
|  | lrc | 0.00 | 79 | 0 |
|  | hd | 57.61 | 79 | 4,551.31 |
|  | dp | 159.68 | 79 | 12,614.60 |
|  | hsp | 2,686.92 | 79 | 212,266.92 |
|  | er | 494.63 | 79 | 39,075.83 |
|  | ha | 5,543.66 | 79 | 437,948.90 |
|  | ihc | 466.85 | 79 | 36,880.85 |
|  | ops | 1,611.23 | 79 | 127,287.29 |

md indicates medical devices; lrc, local residential care; hd, hospital drugs; dp, drug prescriptions; hsp, hospice; er, emergency room; ha, hospital admission; ihc, integrated home care; ops, outpatient services.
