# Supplementary material for: Cost profiles of cancer patients at the end of life: Estimates from the EPICOST-study
Source: PLoS One. 2025 Feb 14;20(2):e0318849. doi: 10.1371/journal.pone.0318849 (PMC11828425; doi:10.1371/journal.pone.0318849)
Supplement: S2 Table — (DOCX) [file pone.0318849.s002.docx]

**S2 Table. Descriptive statistics of the average monthly cost in the final phase by sex, cancer type and health care services database.**

|  | **Mean** | **Median** | **Minimum** | **Maximum** | **Standard Deviation** | **P-value** |
| --- | --- | --- | --- | --- | --- | --- |
| **Cancer type: colon**  **Health care services database: hd** | | | | | | 0.033^a^ |
| **Male** | 285.32 | 332.87 | 28.91 | 363.44 | 103.90 |  |
| **Female** | 201.91 | 212.20 | 41.81 | 361.14 | 81.85 |  |
| **Cancer type: colon**  **Health care services database: ops** | | | | | | 0.017^b^ |
| **Male** | 271.55 | 271.61 | 43.28 | 409.57 | 100.81 |  |
| **Female** | 180.76 | 182.65 | 46.18 | 303.65 | 66.23 |  |

^a^ Nonparametric Mann-Whitney U test

^b^ Parametric T-test

hd indicates hospital drugs; ops, outpatient services.
