# Supplementary material for: Cost profiles of cancer patients at the end of life: Estimates from the EPICOST-study
Source: PLoS One. 2025 Feb 14;20(2):e0318849. doi: 10.1371/journal.pone.0318849 (PMC11828425; doi:10.1371/journal.pone.0318849)
Supplement: S3 Table — (DOCX) [file pone.0318849.s003.docx]

**S3 Table. Descriptive statistics of the average monthly cost in the final phase by age group, cancer type and health care services database.**

|  | **Mean** | **Median** | **Minimum** | **Maximum** | **Standard Deviation** | **P-value** |
| --- | --- | --- | --- | --- | --- | --- |
| **Cancer type: rectum**  **Health care services database: all** | | | | | | <0.001^a^ |
| **15-49 y** | 250.78 | 0.00 | 0.00 | 2,835.00 | 515.66 |  |
| **50-69 y** | 244.66 | 79.19 | 0.00 | 1,978.31 | 344.68 |  |
| **70+ y** | 146.89 | 62.32 | 0.00 | 1,836.58 | 239.62 |  |
| **Cancer type: melanoma**  **Health care services database: all** | | | | | | 0.030^b^ |
| **15-49 y** | 471.85 | 4.01 | 0.00 | 5,671.99 | 1,086.98 |  |
| **50-69 y** | 324.09 | 33.43 | 0.00 | 3,538.00 | 680.29 |  |
| **70+ y** | 154.92 | 24.55 | 0.00 | 1,506.26 | 292.84 |  |
| **Cancer type: colon**  **Health care services database: hd** | | | | | | 0.001^c^ |
| **15-49 y** | 2,778.32 | 2,817.58 | 1.76 | 5,671.99 | 1,518.99 |  |
| **50-69 y** | 1,715.56 | 1,521.69 | 546.18 | 3,538.00 | 813.49 |  |
| **70+ y** | 608.44 | 655.27 | 85.46 | 857.93 | 205.22 |  |
| **Cancer type: colon**  **Health care services database: ops** | | | | | | 0.007^d^ |
| **15-49 y** | 59.34 | 0.00 | 0.00 | 700.00 | 201.77 |  |
| **50-69 y** | 109.59 | 80.86 | 0.00 | 279.98 | 117.51 |  |
| **70+ y** | 22.39 | 1.13 | 0.45 | 184.65 | 53.78 |  |
| **Cancer type: breast**  **Health care services database: hd** | | | | | | <0.001^e^ |
| **15-49 y** | 796.87 | 844.97 | 225.79 | 1,082.69 | 226.20 |  |
| **50-69 y** | 364.36 | 370.32 | 56.87 | 569.49 | 134.04 |  |
| **70+ y** | 72.35 | 76.37 | 29.22 | 103.37 | 19.77 |  |
| **Cancer type: breast**  **Health care services database: dp** | | | | | | <0.001^f^ |
| **15-49 y** | 101.36 | 87.95 | 71.27 | 197.21 | 38.03 |  |
| **50-69 y** | 45.53 | 37.99 | 25.77 | 83.37 | 18.29 |  |
| **70+ y** | 29.42 | 28.22 | 24.33 | 39.39 | 4.44 |  |
| **Cancer type: breast**  **Health care services database: ops** | | | | | | <0.001^g^ |
| **15-49 y** | 321.84 | 270.98 | 157.20 | 592.05 | 120.76 |  |
| **50-69 y** | 220.74 | 214.72 | 126.24 | 302.56 | 49.24 |  |
| **70+ y** | 80.93 | 82.85 | 29.71 | 141.12 | 26.35 |  |
| **Cancer type: melanoma**  **Health care services database: hd** | | | | | | <0.001^h^ |
| **15-49 y** | 2,778.32 | 2,817.58 | 1.76 | 5,671.99 | 1,518.99 |  |
| **50-69 y** | 1,715.56 | 1,521.69 | 546.18 | 3,538.00 | 813.49 |  |
| **70+ y** | 608.44 | 655.27 | 85.46 | 857.93 | 205.22 |  |
| **Cancer type: melanoma**  **Health care services database: dp** | | | | | | 0.004^i^ |
| **15-49 y** | 11.45 | 8.45 | 3.17 | 44.43 | 11.19 |  |
| **50-69 y** | 22.45 | 20.81 | 7.35 | 59.42 | 13.65 |  |
| **70+ y** | 23.35 | 17.78 | 9.90 | 57.45 | 15.29 |  |
| **Cancer type: melanoma**  **Health care services database: ops** | | | | | | 0.020^j^ |
| **15-49 y** | 59.34 | 0.00 | 0.00 | 700.00 | 201.77 |  |
| **50-69 y** | 109.59 | 80.86 | 0.00 | 279.98 | 117.51 |  |
| **70+ y** | 22.39 | 1.13 | 0.45 | 184.65 | 53.78 |  |
| **Cancer type: rectum**  **Health care services database: hd** | | | | | | 0.013^k^ |
| **15-49 y** | 741.48 | 976.59 | 0.00 | 1260.53 | 547.63 |  |
| **50-69 y** | 515.18 | 615.44 | 51.93 | 745.40 | 209.11 |  |
| **70+ y** | 161.57 | 182.37 | 27.76 | 241.01 | 62.07 |  |
| **Cancer type: rectum**  **Health care services database: dp** | | | | | | 0.011^l^ |
| **15-49 y** | 93.13 | 86.70 | 0.00 | 294.93 | 97.59 |  |
| **50-69 y** | 75.27 | 72.05 | 40.06 | 174.09 | 37.57 |  |
| **70+ y** | 23.11 | 21.14 | 9.30 | 40.88 | 10.80 |  |
| **Cancer type: rectum**  **Health care services database: ops** | | | | | | <0.001^m^ |
| **15-49 y** | 709.73 | 746.40 | 16.25 | 1860.28 | 482.36 |  |
| **50-69 y** | 372.97 | 367.30 | 124.92 | 500.98 | 102.46 |  |
| **70+ y** | 166.62 | 168.66 | 24.92 | 277.98 | 75.31 |  |

^a^ Nonparametric Kruskal-Wallis H test, pairwise comparison using Dunn’s Test with Bonferroni adjustment. Statistical significant difference between 15-49 y and 50-69 y (p-value <0.001), and between 15-49 y and 70+ y (p-value <0.001).

^b^ Nonparametric Kruskal-Wallis H test, pairwise comparison using Dunn’s Test with Bonferroni adjustment. Statistical significant difference between 15-49 y and 50-69 y (p-value equal to 0.047).

^c^ Nonparametric Kruskal-Wallis H test, pairwise comparison using Dunn’s Test with Bonferroni adjustment. Statistical significant difference between 15-49 y and 70+ y (p-value equal to 0.008), and between 50-69 y and 70+ y (p-value equal to 0.003).

^d^ Nonparametric Kruskal-Wallis H test, pairwise comparison using Dunn’s Test with Bonferroni adjustment. Statistical significant difference between 50-69 y and 70+ y (p-value equal to 0.005).

^e^ Nonparametric Kruskal-Wallis H test, pairwise comparison using Dunn’s Test with Bonferroni adjustment. Statistical significant difference between 15-49 y and 50-69 y (p-value equal to 0.028), between 15-49 y and 70+ y (p-value <0.001), and between 50-69 y and 70+ y (p-value equal to 0.02828).

^f^ Nonparametric Kruskal-Wallis H test, pairwise comparison using Dunn’s Test with Bonferroni adjustment. Statistical significant difference between 15-49 y and 50-69 y (p-value equal to 0.008), and between 15-49 y and 70+ y (p-value <0.001).

^g^ Nonparametric Kruskal-Wallis H test, pairwise comparison using Dunn’s Test with Bonferroni adjustment. Statistical significant difference between 15-49 y and 70+ y (p-value <0.001), and between 50-69 y and 70+ y (p-value equal to 0.002).

^h^ Nonparametric Kruskal-Wallis H test, pairwise comparison using Dunn’s Test with Bonferroni adjustment. Statistical significant difference between 15-49 y and 70+ y (p-value <0.001), and between 50-69 y and 70+ y (p-value equal to 0.010).

^i^ Nonparametric Kruskal-Wallis H test, pairwise comparison using Dunn’s Test with Bonferroni adjustment. Statistical significant difference between 15-49 y and 50-69 y (p-value equal to 0.012), and between 15-49 y and 70+ y (p-value equal to 0.014).

^j^ Nonparametric Kruskal-Wallis H test, pairwise comparison using Dunn’s Test with Bonferroni adjustment. Statistical significant difference between 15-49 y and 50-69 y (p-value equal to 0.017).

^k^ Nonparametric Kruskal-Wallis H test, pairwise comparison using Dunn’s Test with Bonferroni adjustment. Statistical significant difference between 15-49 y and 50-69 y (p-value equal to 0.017).

^l^ Nonparametric Kruskal-Wallis H test, pairwise comparison using Dunn’s Test with Bonferroni adjustment. Statistical significant difference between 50-69 y and 70+ y (p-value equal to 0.008).

^m^ Nonparametric Kruskal-Wallis H test, pairwise comparison using Dunn’s Test with Bonferroni adjustment. Statistical significant difference between 15-49 y and 70+ y (p-value <0.001), and between 50-69 y and 70+ y (p-value equal to 0.032).

hd indicates hospital drugs; ops, outpatient services; dp, drug prescriptions.
