# Supplementary material for: Cost profiles of cancer patients at the end of life: Estimates from the EPICOST-study
Source: PLoS One. 2025 Feb 14;20(2):e0318849. doi: 10.1371/journal.pone.0318849 (PMC11828425; doi:10.1371/journal.pone.0318849)
Supplement: Attachment — Submitted filename: Comments.docx [file pone.0318849.s004.docx]

The manuscript is well written, it faces the important issue of cancer costs in EOL and it has a robust approach in treating it.

A clarification is needed on the Discussion paragraph (line 350-354) where the contributions to total health expenditures are mentioned.

Please clarify to what table/figure the percentages refer and verify if there is a mistake on the percentage of colon cancer cost.
